# Group V Secretory Phospholipase A_2_ Is Involved in Tubular Integrity and Sodium Handling in the Kidney

**DOI:** 10.1371/journal.pone.0147785

**Published:** 2016-01-28

**Authors:** João Luiz Silva-Filho, Diogo Barros Peruchetti, Felipe Moraes-Santos, Sharon Schilling Landgraf, Leandro Souza Silva, Gabriela Modenesi Sirtoli, Daniel Zamith-Miranda, Christina Maeda Takiya, Ana Acacia Sá Pinheiro, Bruno Lourenço Diaz, Celso Caruso-Neves

**Affiliations:** 1 Instituto de Biofísica Carlos Chagas Filho, Universidade Federal do Rio de Janeiro, Rio de Janeiro, RJ, Brazil; 2 Instituto Federal de Educação, Ciência e Tecnologia, Rio de Janeiro, RJ, Brazil; 3 Instituto de Ciências Biomédicas, Universidade Federal do Rio de Janeiro, Rio de Janeiro, RJ, Brazil; 4 Instituto Nacional para Pesquisa Translacional em Saúde e Ambiente na Região Amazônica, Conselho Nacional de Desenvolvimento Científico e Tecnológico/MCT, Rio de Janeiro, RJ, Brazil; 5 Instituto Nacional de Ciência e Tecnologia em Biologia e Bioimagem, Rio de Janeiro, RJ, Brazil; Center for Molecular Biotechnology, ITALY

## Abstract

Group V (GV) phospholipase A_2_ (PLA_2_) is a member of the family of secreted PLA_2_ (sPLA2) enzymes_._ This enzyme has been identified in several organs, including the kidney. However, the physiologic role of GV sPLA_2_ in the maintenance of renal function remains unclear. We used mice lacking the gene encoding GV sPLA_2_ (*Pla2g5*^*−/−*^) and wild-type breeding pairs in the experiments. Mice were individually housed in metabolic cages and 48-h urine was collected for biochemical assays. Kidney samples were evaluated for glomerular morphology, renal fibrosis, and expression/activity of the (Na^+^ + K^+^)-ATPase α1 subunit. We observed that plasma creatinine levels were increased in *Pla2g5*^*−/−*^ mice following by a decrease in creatinine clearance. The levels of urinary protein were higher in *Pla2g5*^*−/−*^ mice than in the control group. Markers of tubular integrity and function such as γ-glutamyl transpeptidase, lactate dehydrogenase, and sodium excretion fraction (FE_Na_^+^) were also increased in *Pla2g5*^*−/−*^ mice. The increased FE_Na_^+^ observed in *Pla2g5*^*−/−*^ mice was correlated to alterations in cortical (Na^+^ + K^+^) ATPase activity/ expression. In addition, the kidney from *Pla2g5*^*−/−*^ mice showed accumulation of matrix in corticomedullary glomeruli and tubulointerstitial fibrosis. These data suggest GV sPLA_2_ is involved in the maintenance of tubular cell function and integrity, promoting sodium retention through increased cortical (Na^+^ + K^+^)-ATPase expression and activity.

## Introduction

Phospholipase A_2_ (PLA_2_) is a superfamily of enzymes that provides free fatty acids and lysophospholipids from the hydrolysis of the ester bond at the *sn*-2 position of glycerophospholipids [1]. Currently, PLA_2_ enzymes have been classified into 6 types with 16 distinct groups according to their structure, function, and cellular location [1].

Secretory phospholipase A_2_ (sPLA_2_) was the first type of PLA_2_ discovered [2]. In mammals, this family contains 10 catalytically active isoforms, including the group V sPLA_2_ (GV sPLA_2_) [2]. GV sPLA_2_ plays an important role in diverse biological and pathological cellular process due to its capacity to metabolize phospholipids and produce a free fatty acid and a lysophospholipid. Although it has been reported that GV sPLA2 promotes the release of arachidonic acid and subsequent generation of eicosanoids, such as prostaglandins and leukotrienes [1,2], this may not be relevant to its physiologic functions [3,4]. However, GV sPLA2 may still participate in the synthesis of eicosanoid through activation of GIVA PLA_2_ [5–7] or induction of cyclooxygenase (COX)-2 activity [8,9].

Several animal studies have revealed that GV sPLA_2_ contributes to eosinophilic pulmonary inflammation [10–12], abdominal aortic aneurysms [13], ischemic injury [14], and autoimmune diseases [15]. In addition, GV sPLA_2_ has also shown potent antibacterial and antiviral properties [16]. In this regard, several studies have elucidated the role of GV sPLA_2_ in different systems, particularly in pathological processes, but the function of this enzyme in the kidney, regulation of renal hemodynamics or involvement in kidney disease, remains unclear.

Expression of GV sPLA_2_ has been demonstrated in the kidney of rats [17] and mice [18]. It has also been shown that GV sPLA_2_ is constitutively expressed in the tubular epithelium of normal human kidneys and its expression is markedly upregulated in the tubules and glomeruli during kidney damage [14]. Studies on human embryonic kidney 293 cells (HEK293) and in primary cultures of mouse mesangial cells have also shown that GV sPLA_2_ amplifies the release and conversion of arachidonic acid into prostaglandins by increasing GIVA PLA_2_ and COX-2 activity [19,20]. However, the in vivo significance of the activity of GV sPLA_2_ on renal function has not been described.

In the present work, we used mice with a homozygous disruption in the gene encoding GV sPLA_2_ (*Pla2g5*^*−/−*^) to clarify the role of this PLA_2_ group on renal function. Our data revealed that GV sPLA_2_ plays a physiologic role in the maintenance of renal function and sodium handling, with a major influence on the tubular compartment rather than in the glomerulus.

## Materials and Methods

### Animals

Mice with targeted disruption of the gene encoding GV sPLA_2_ (*Pla2g-5*^*−/−*^) were generated by Satake et al. [21]. We used 12-week-old male *Pla2g5*-null and wild-type (WT) mice in a C57BL/6 genetic background in all experiments. Mice were caged with free access to food and fresh water in a temperature-controlled room (22–24°C) with a 12-h light/dark cycle until used. This study was carried out in strict accordance with the recommendations in the *Guide for the Care and Use of Laboratory Animals* of the National Institutes of Health. The protocol was approved by the Institutional Ethics Committee of Federal University of Rio de Janeiro (permit number IBCCF004). For the euthanasia procedure, animals were anesthetized with ketamine (80 mg/kg body weight) and xylazine (5 mg/kg body weight) before blood collection via cardiac puncture.

### Reverse transcription-polymerase chain reaction

Kidneys of WT and *Pla2g5*^*−/−*^ mice were dissected and total RNA from the renal cortex and medulla was extracted using TRIZOL reagent (Invitrogen, Karlsruhe, Germany). Contaminating genomic DNA was removed by DNase I (Fermentas, St. Leon-Rot, Germany) before reverse transcription (RT) of 1 μg of total RNA using a Superscript III kit (Invitrogen, Karlsruhe, Germany). To determine the expression of GV sPLA_2_ in mice kidneys, cDNA was submitted to conventional polymerase chain reaction (PCR) using the following primers: forward AAC AGG CGC TGA GAC CAG, and reverse GAC ATT AGC AGA GGA AGT TGG G and settings: denaturation—95°C, annealing—53°C and extension – 72°C in a 35 cycles PCR reaction. The amplicons generated were resolved on agarose gel electrophoresis and analyzed under UV light. A band of the expected size (~455 bp) for GV sPLA_2_ mRNA was observed in the cortex and medulla of kidneys obtained from WT mice [22]. On the other hand, RT-PCR analysis of *Pla2g5*^*−/−*^ mice confirmed the lack of GV sPLA_2_ mRNA in these animals (data not shown).

### Measurement of renal function

Mice were kept individually in metabolic cages to analyze renal function. The cages were maintained in a temperature-controlled room (22–24°C) with a 12-h light/dark cycle, with free access to tap water and standard rodent diet. After 2 days of acclimatization, 48-h urine was collected to determine urine volume, total protein, creatinine, sodium, γ-glutamyl transpeptidase (γGT), and lactate dehydrogenase (LDH) concentrations. Before analysis, urine samples were centrifuged at 3000×*g* for 10 min to clear sediments. Blood samples were collected and centrifuged at 1200×*g* for 10 min at 4°C to obtain plasma to measure sodium and creatinine concentrations.

The levels of urinary protein were determined by the pyragallol red method (Gold Analisa kit #498M, Belo Horizonte, MG, Brazil) and creatinine by the alkaline picrate method (Gold Analisa kit #335, Belo Horizonte, MG, Brazil). Kits for γGT (Bioclin kit #K080, Belo Horizonte, MG, Brazil) and LDH (Gold Analisa kit #457, Belo Horizonte, MG, Brazil) were used for quantitative determination of the enzyme activity. Sodium levels were analyzed by the photometric colorimetric test (Human Diagnostics Worldwide kit #573351, Wiesbaden, Germany). Plasma and urine osmolality were measured on an Advanced Micro Sample Osmometer 3320 (Advanced Instruments, Norwood, MA).

### Histologic and histomorphometric studies

Kidneys were fixed in a 4% buffered formalin solution and embedded in paraffin. Histologic sections (3-μm thick) of kidney were obtained and stained with periodic acid-Schiff reagent (PAS; Sigma-Aldrich, St Louis, MA) for analysis of the mesangial surface of subcapsular and corticomedullary glomeruli. In addition, 7-μm-thick sections were cut to assess the deposition of collagen fibers with Picrosirius Red staining (Sigma-Aldrich, St. Louis, MA). Only interstitial collagen was counted, and vessels and glomeruli were excluded. Data were expressed as a percentage of the interstitial area with positive staining. Quantification analysis of PAS and Picrosirius Red-stained sections were performed using Image-Pro Plus analysis software on 25 photomicrographs in a light microscope equipped with a camera (Eclipse E800, Nikon).

### Preparation of the homogenate fraction

The homogenate fraction of the renal cortex and medulla was obtained as described previously [23]. Briefly, kidneys were removed and homogenized in a cold solution containing 250 mmol/l sucrose, 10 mmol/l HEPES–Tris (pH 7.6), 2 mmol/l EDTA, and 1 mmol/l phenylmethylsulfonyl fluoride. Homogenates were centrifuged at 7000×*g* at 4°C for 10 min and the final supernatant was stored at –80°C. Protein concentrations were determined by the Folin phenol method [24] using bovine serum albumin as standard.

### Immunoblotting

Proteins were resolved on sodium dodecyl sulfate-polyacrylamide gels and transferred to nitrocellulose membrane (Millipore Corporation, Bellerica, MA), according to the manufacturer's instructions. The (Na^+^ + K^+^)-ATPase α1 subunit was immunodetected in the homogenate fraction of the renal cortex and medulla with specific primary antibody (1:10 000; #05–369, Millipore Corporation, Bellerica, MA). After antibody labeling, detection was performed with ECL-plus (Amersham Biosciences, Piscataway, NJ).

### Measurement of (Na^+^ + K^+^)-ATPase activity

ATPase activity was evaluated by spectrophotometric measurement of inorganic phosphate released from ATP with the use of ammonium molybdate as described by Maritno et al. [25]. The composition of the standard assay medium was 15 mM MgCl_2_, 5 mM ATP·Na^+^-Tris (pH 7.0), 150 mM NaCl, and 15 mM KCl. The reaction was started by the addition of 3 mg/ml protein from the renal cortex or medulla homogenate (final concentration, 0.3 mg/ml). After 10 min of incubation at 37°C, the reaction was stopped by the addition of trichloroacetic acid. Phosphate solutions (0–40 μM) were used as standards. The phosphate content was determined by measurement of absorbance at 660 nm. The (Na^+^ + K^+^)-ATPase activity was calculated as the difference in ATPase activity between renal cortex or medulla homogenate exposed to ouabain and those not exposed.

### Statistical analysis

Each experiment was carried out using 4 animals per group. Data are reported as the mean ± standard error of at least 2 representative experiments. Statistical analysis was performed using Prism software (GraphPad Software, version 5), and, unless otherwise stated, means were compared by the two-tailed Student t test. The significance level was set at α = 0.05.

## Results

### GV sPLA_2_ is important to renal function homeostasis

To elucidate the physiologic role of GV sPLA_2_ on renal function, we measured related parameters in WT and *Pla2g5*^*−/−*^ mice (Table 1). The results show that urinary flow and creatinine clearance (CCr, a marker of glomerular flow rate) were decreased in *Pla2g5*^*−/−*^ mice compared with the WT group (Table 1). The decrease in CCr was followed by an increase in plasma creatinine in *Pla2g5*^*−/−*^ mice. Urinary osmolality (U_osm_) was increased in *Pla2g5*^*−/−*^ mice without changes in plasma osmolality (P_osm_). Body weight was not changed in both WT and *Pla2g5*^*−/−*^ mice. The ratio of urinary protein to creatinine (UPCr), a marker of renal injury [26], was slightly higher in *Pla2g5*^*−/−*^ mice compared with WT animals. These results indicate that GV sPLA_2_ is important for the maintenance of renal function.

**Table 1:** Renal Function Parameters.

	Wild-type (n = 18)	*Pla2g5*^*-/-*^ (n = 36)
**Body Weight (g)**	22.95 ± 0.54	23.4 ± 0.37
**48h Urinary Flow (x10**^**-3**^ **mL/min)**	0.89 ± 0.05	0.57 ± 0.03*
**Urinary Creatinine (mg/dL)**	48.36 ± 3.79	52.4 ± 2.88
**Plasma Creatinine (x10**^**-1**^ **mg/dL)**	0.68 ± 0.06	1.29 ± 0.11*
**Creatinine Clearance (mL/min)**	0.65 ± 0.05	0.28 ± 0.03*
**Urinary Osmolality (mOsm/KgH**_**2**_**O)**	2784.3 ± 193.35	3815.0 ± 391.15*
**Plasma Osmolality (mOsm/KgH**_**2**_**O)**	338.67 ± 7.75	339.00 ± 6.24
**UPCr**	0.74 ± 0.12	1.20 ± 0.12*

*Statistically significant in relation to *WT mice (p<0.05); UPCr: ratio between urinary protein and creatinine

### Mild glomerular morphologic changes in Pla2g5^−/−^ mice

**Fig 1:**
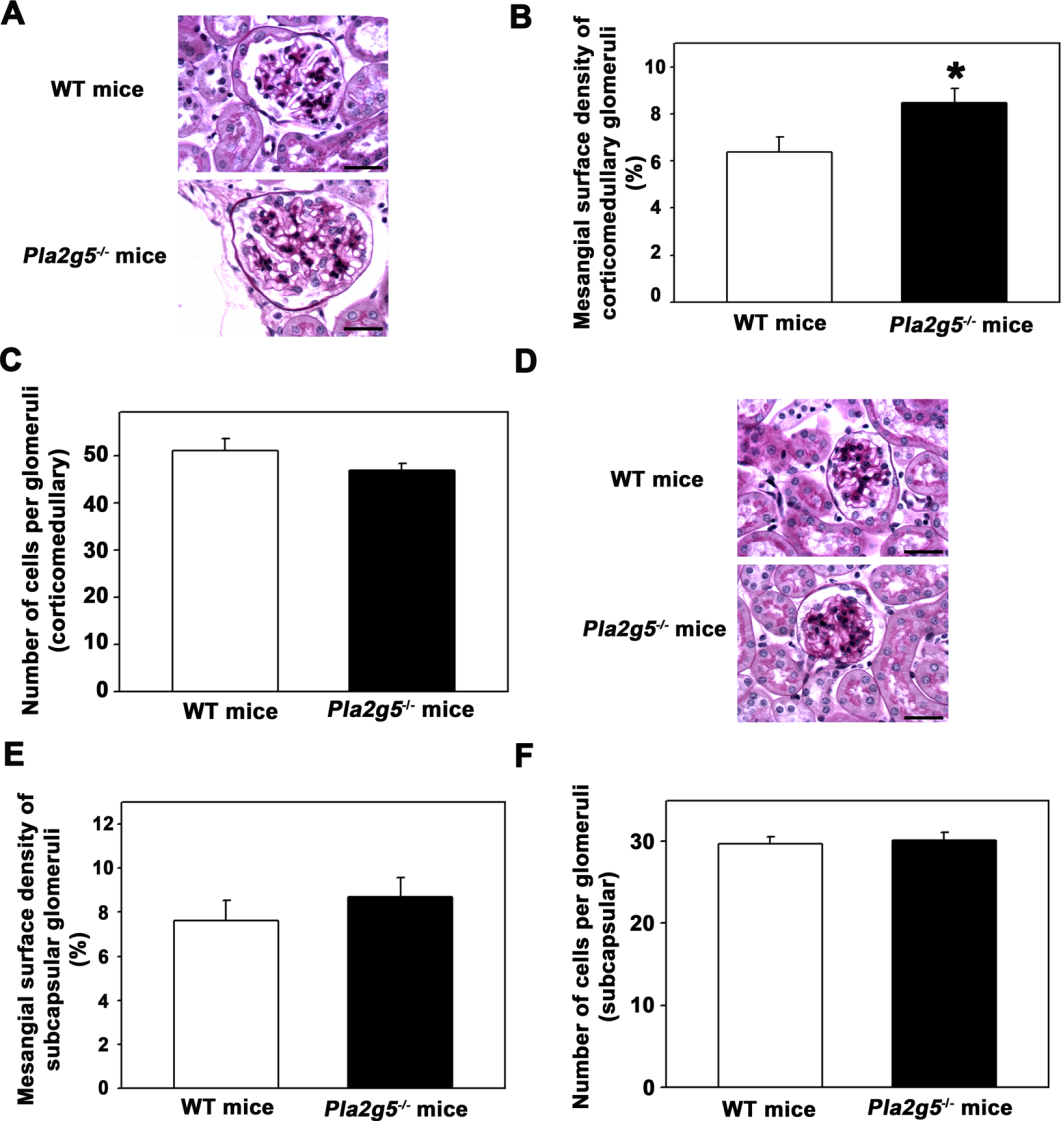
Mild glomerular morphologic changes are observed in Pla2g5^−/−^ mice. PAS reagent was used for analysis of the mesangial surface of corticomedullary (A, B) and subcapsular glomeruli (C, D), as described in the Materials and Methods. Representative photomicrographs (magnification 40×) of (A) the corticomedullary glomerulus and (C) the subcapsular glomerulus. (B) Quantitative analysis of the corticomedullary and (D) subcapsular glomeruli (n = 6 per group). The results are expressed as means ± SE. *Statistically significant in relation to WT mice (P < 0.05).

Several studies have shown that a decline in the glomerular filtration rate can be correlated with glomerular morphologic changes [27]. Thus, we wondered whether the decreased CCr in *Pla2g5*^*−/−*^ mice is correlated to morphologic changes in the glomerulus. We analyzed the glomerular structure of WT and *Pla2g5*^*−/−*^ mice. The subcapsular and corticomedullary glomeruli of WT and *Pla2g5*^*−/−*^ groups were assessed by light microscopy (Fig 1). The mesangial surface was revealed by accumulation of PAS-positive material in mesangial area.

As shown in Fig 1, although the number of cells per glomerulus was not different, the mesangial surface of the corticomedullary glomeruli was increased in *Pla2g5*^*−/−*^ mice compared with controls (Fig 1A–1C). Conversely, the mesangial surface of the subcapsular glomeruli and cellularity were not significantly different between the WT and *Pla2g5*^*−/−*^ groups (Fig 1D–1F). Thus, these results indicate that changes in the CCr, observed in *Pla2g5*^*−/−*^ mice, are not correlated to major glomerular morphologic alterations in these mice and may be only caused by changes in glomerular function.

### GV sPLA_2_ is critical for the maintenance of tubular integrity

It is well known that glomerular injury and tubular impairment are involved in early events that lead to proteinuria [26]. Because no major changes in glomerulus structure seem to occur in *Pla2g5*^*−/−*^ mice, we investigated if the higher UPCr observed in these *knock-out* mice could be associated with changes in renal tubular integrity and function.

**Fig 2:**
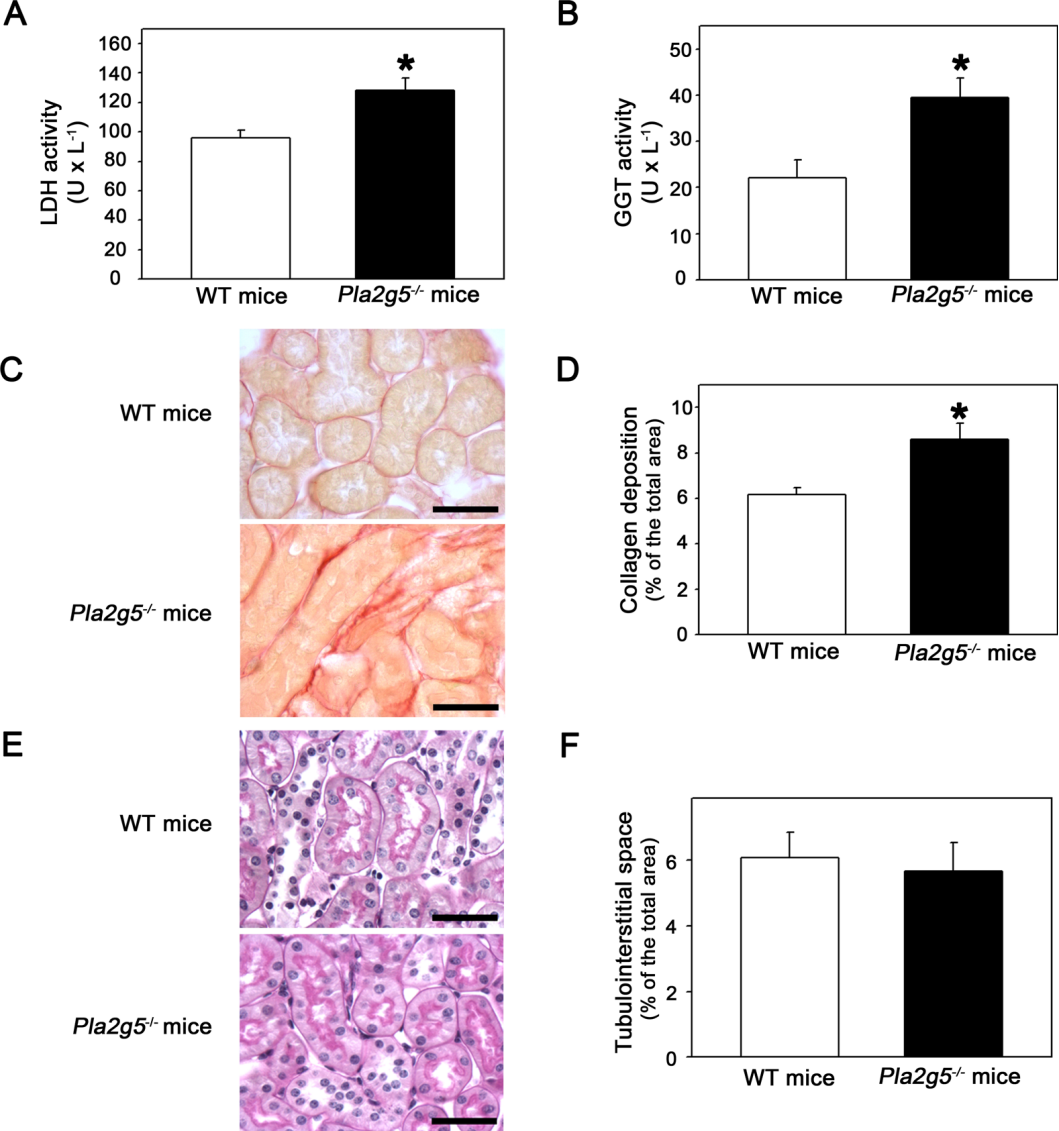
Urinary tubular enzymes and collagen deposition, markers of tubular injury, are increased in *Pla2g-5*^*−/−*^ mice. (A) LDH (B) and γGT activities were measured in urine samples as markers of tubular injury (*n* = 8 per group). Collagen deposition in the renal cortex was visualized by Picrosirius Red staining. (C) Representative photomicrographs (magnification 40×) of collagen deposition in the renal cortex of WT and *Pla2g-5*^*−/−*^ mice. (D) Quantitative analysis of the collagen deposition (*n* = 6 per group). The results are expressed as means ± SE. *Statistically significant in relation to WT mice (*P* < 0.05).

LDH activity and γGT activity, markers of altered tubular integrity, were determined in urine (Fig 2A and 2B), and fibrosis was visualized by Picrosirius Red staining for collagen fibers (Fig 2C–2F). Fig 2A and 2B shows that urinary LDH and γGT activities were significantly increased in *Pla2g5*^*−/−*^ mice in relation to control mice. A similar profile was observed in cortical interstitial fibrosis. Collagen deposition was enhanced in *Pla2g5*^*−/−*^ mice compared with the WT group (Fig 2C and 2D). On the other hand, tubular interstitial space was not changed in the different mice groups (Fig 2E and 2F). These results suggest that GV sPLA_2_ is critical to conserve tubular integrity and the higher proteinuria observed in *Pla2g5*^*−/−*^ mice may be associated with deficiency of this function.

### GV sPLA_2_ promotes sodium retention

**Fig 3:**
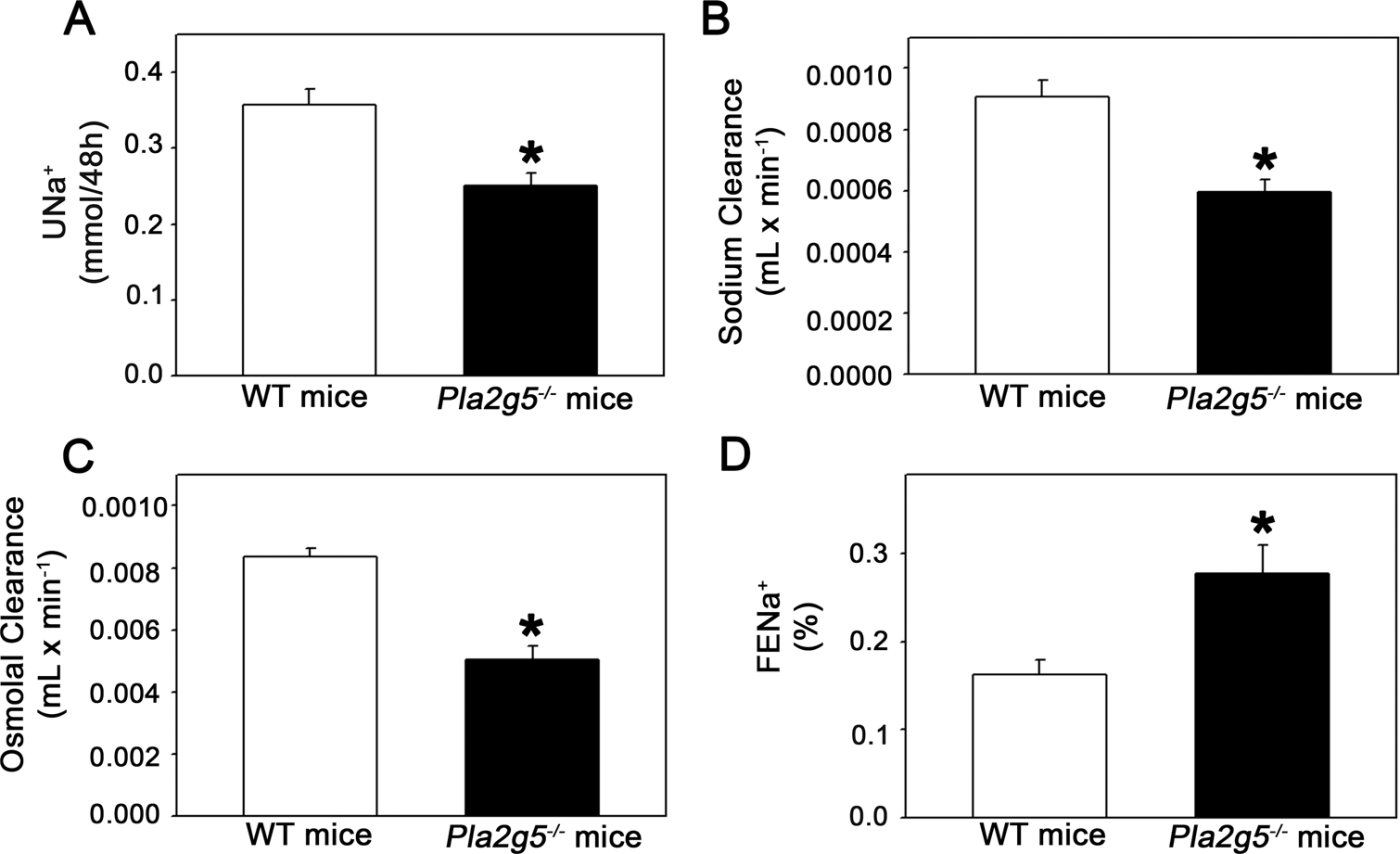
GV sPLA_2_ promotes sodium retention. (A) Urinary sodium excretion (U_Na_^+^V), (B) clearance of sodium (C_Na_^+^), (C) osmolar clearance, and (D) FE_Na_^+^ in WT and Pla2g-5^−/−^ mice. The number of mice analyzed is given in Table 1. The results are expressed as means ± SE. *Statistically significant in relation to WT mice (P < 0.05).

Previous studies have shown a positive correlation between tubular cell damage and the sodium excretion fraction (FE_Na_^+^) [28], indicating impairment of tubular function. Thus, based on the aforementioned results suggesting a critical role of GV sPLA_2_ in the preservation of tubular integrity, we wondered whether this enzyme also affects tubular function. Because sodium handling is a hallmark of the tubular function, we verified some functional parameters related to renal sodium excretion in *Pla2g5*^*−/−*^ and WT mice (Fig 3). Fig 3A and 3B shows that urinary sodium excretion (U_Na_^+^V) and clearance of sodium (C_Na_^+^) were decreased in the *Pla2g5*^*−/−*^ group compared with the control group. In accordance, decreased osmolar clearance (C_osm_) was also observed in *Pla2g5*^*−/−*^ mice (Fig 3C). On the other hand, FE_Na_^+^ was increased in *Pla2g-5*^*/−*^ mice in relation to the WT group (Fig 3D). Thus, besides impairment of tubular integrity, *Pla2g5*^*−/−*^ mice show changes in tubular function, with a consequent higher FE_Na_^+^. These results suggest that besides maintaining tubular integrity, GV sPLA_2_ affects tubular function, such as sodium handling.

### GV sPLA_2_ upregulates activity and expression of cortical (Na^+^ + K^+^)-ATPase

**Fig 4:**
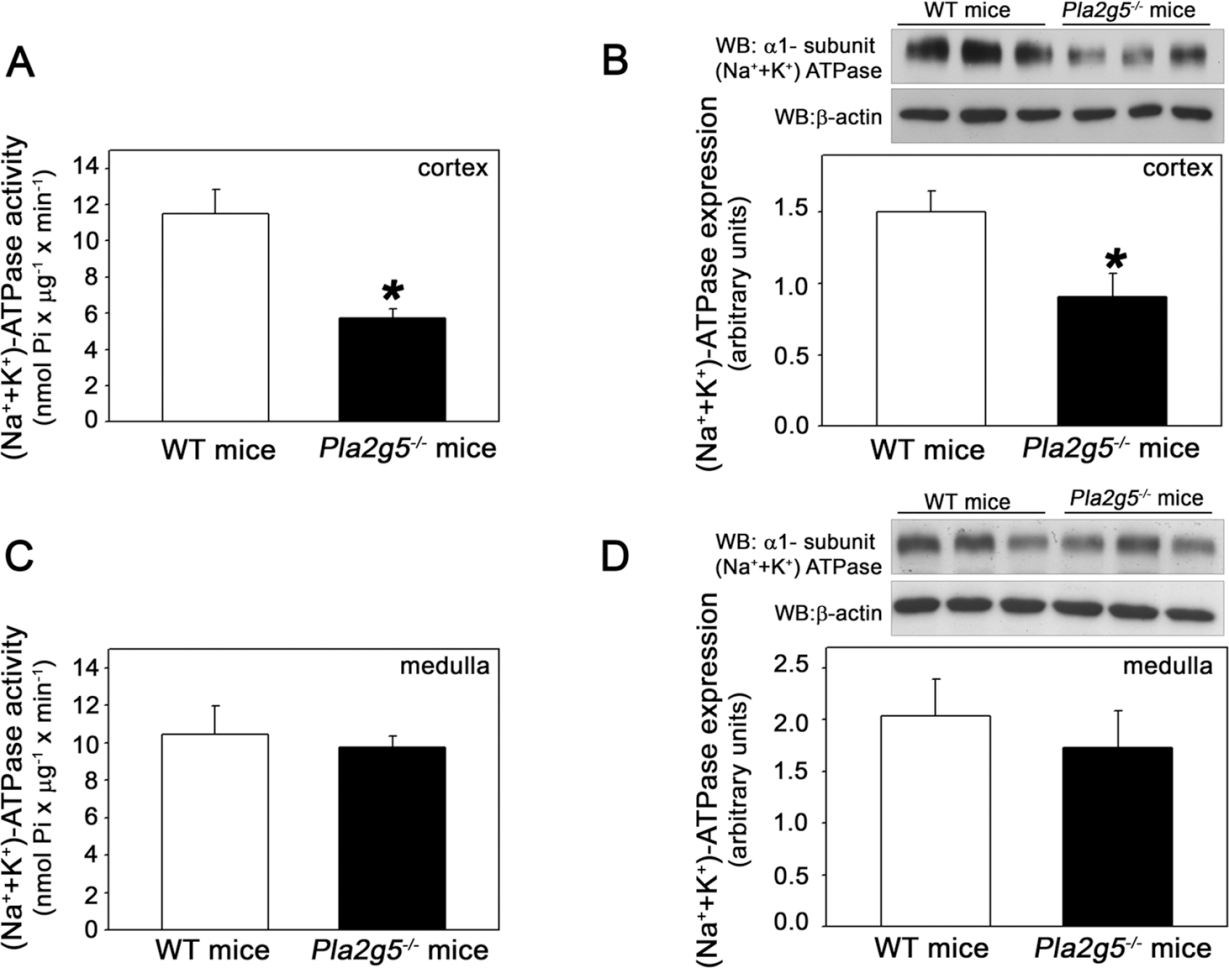
GV sPLA2 upregulates activity and expression of cortical (Na+ + K+)-ATPase. Expression and activity of (Na^+^ + K^+^)-ATPase in WT and *Pla2g-5*^*−/−*^ mice. ATPase activity from the renal cortex (A) and medulla homogenate (C) was determinate by the colorimetric method. Immunoblotting was performed for the (Na^+^ + K^+^)-ATPase α1 subunit in (B) the renal cortex and (D) the medullar preparation of both WT and *Pla2g5*^*−/−*^ mice, as described in the Materials and Methods (*n* = 8 per group). The results are expressed as means ± SE. *Statistically significant in relation to WT mice (*P* < 0.05).

The sodium pump (Na^+^ + K^+^)-ATPase is one of the principal determinants of tubular sodium transport [29]. Therefore, in this experimental group, we investigated whether the increase in FE_Na_^+^ observed in *Pla2g5*^*−/−*^ mice could be correlated with alterations in (Na^+^ + K^+^)-ATPase. Activity and α1 subunit expression of (Na^+^ + K^+^)-ATPase were evaluated in the renal cortex and medullar preparations of both WT and *Pla2g5*^*−/−*^ mice. Cortical (Na^+^ + K^+^)-ATPase activity was decreased in the *Pla2g5*^*−/−*^ group compared with the control group (Fig 4A). In agreement, cortical expression of the α1 subunit of (Na^+^ + K^+^)-ATPase was decreased in *Pla2g5*^*−/−*^ mice (Fig 4B). However, no significant differences were observed in medullar (Na^+^ + K^+^)-ATPase activity and α1 expression between the *Pla2g5*^*−/−*^ and WT groups (Fig 4C and 4D). These observations of decreased activity and expression of cortical (Na^+^ + K^+^)-ATPase could explain the increased FE_Na_^+^ in *Pla2g5*^*−/−*^ mice. Therefore, GV sPLA_2_ is important for tubular function, inducing sodium retention by increasing the activity and expression of cortical (Na^+^ + K^+^)-ATPase.

## Discussion

GV sPLA_2_ is a highly expressed enzyme in mouse and human heart and placenta [14,18], but GV sPLA_2_ mRNA was also detected to a lesser extent in mouse kidney [18]. GV sPLA_2_ belongs to the sPLA_2_ family and it is important in phospholipid metabolism and eicosanoid production [4,11]. This enzyme regulates inflammatory processes [10–12,30,31] and is involved in different pathologies [13–16]. However, the role of GV sPLA_2_ in renal tissue integrity and function has not been described.

Here, we showed renal expression of GV sPLA_2_ and involvement in the maintenance of renal function and sodium handling, revealing new functions for this particular sPLA_2_ group. We used mice with targeted disruption of the *Pla2g5* gene, and confirmed the absence of *Pla2g5* mRNA in *Pla2g5*^*−/−*^ mice by RT-PCR. Mice lacking GV sPLA2 expression can be used to address the physiologic role of this enzyme in different tissues [32], because the similar structural and functional features among different sPLA2 isoenzymes make the development of compounds that selectively inhibit GV sPLA2 enzymes difficult [1,11,32].

GV sPLA2 controls, at least in part, the biosynthesis of leukotrienes (LTs) and prostaglandins (PG) derived from membrane phospholipids, but the molecular mechanisms involved and the location of action of sPLA2 are not totally clear yet [3–7,11,21, 33]. Nevertheless, it has been shown that after being secreted to the extracellular medium, sPLA2 enzymes hydrolyze phospholipids at the outer cellular surface [33]. In parallel, sPLA2 enzymes are reinternalized and localized with COX-2 in the perinuclear membrane, ready to promote the conversion of arachidonic acid into eicosanoids [33–39]. LTs and PGs are implicated in many physiologic functions as well as pathologic conditions in different organs, including the kidney [40,41]. Recently, Kvirkvelia et al. [42] showed that PGE_2_ promotes cellular recovery of established nephritis in mice, modulating podocyte ultrastructure and foot processes and decreasing proteinuria. In our study, mild glomerular morphologic changes and increased urinary protein excretion were observed in *Pla2g5*^*−/−*^ mice. These processes are likely linked to a decrease in PGE_2_ generation in *Pla2g5*^*−/−*^ mice. A 50% lower production of PGE_2_ and LTC_4_ in macrophages as well as reduced COX-2 expression in bone marrow-derived mast cells from *Pla2g5-null* mice compared with control mice has already been demonstrated [8,21]. In addition, GV sPLA_2_ transfection into HEK293 cells induces expression of COX-2, which is the major enzyme involved in the initial conversion of arachidonic acid to prostanoids, such as PGE_2_, in the kidney [43,44].

Although *Pla2g5*^*−/−*^ mice showed decreased CCr along with increased urinary protein excretion, there were no profound changes in glomerular structure. However, more prominent tubular changes were observed, suggesting that GV sPLA_2_ plays a more pronounced role in the function and integrity of the tubular compartment. Previous studies reported that urinary levels of LDH and γGT are linked to apoptosis of tubular renal cells and, consequently, early diagnosis of kidney disease [45]. Here, we verified intense leakage of LDH and γGT in the urine of *Pla2g5*^*−/−*^ mice, which suggests a potential role for GV sPLA_2_ in the integrity of tubular cells. In agreement, Murakami et al. [43] showed that GV sPLA_2_ is important to cell membrane integrity in HEK293 cells.

Changes in the integrity of tubular cells can induce cell dysfunction, which impairs tubular transport and reabsorption mechanisms, leading to decreased protein reabsorption and proteinuria [26,27,46]. In addition, injury to tubular cells can cause cell dedifferentiation and local inflammation leading to increased renal fibrosis [26,27,40]. Thus, alterations in tubular integrity due to the lack of GV sPLA_2_ expression could explain the increased proteinuria and cortical interstitial fibrosis verified in *Pla2g5*^*−/−*^ mice. On the other hand, the higher levels of protein in the tubular lumen act in a positive-feedback manner, further promoting apoptosis of tubular cells and interstitial fibrosis [26,47,48].

Another indication of the status of tubular integrity and function is the FE_Na_^+^, which represents the percentage of sodium filtered by the kidney that is excreted in the urine after tubular handling. Therefore, changes in this parameter represent changes in tubular function and damage [28]. The FE_Na_^+^ was increased in *Pla2g5*^*−/−*^ mice and this phenomenon was correlated to reduced expression and activity of cortical (Na^+^ + K^+^)-ATPase in these animals. These observations further support the importance of GV sPLA_2_ in tubular integrity and function.

With regard to tubular sodium handling by (Na^+^ + K^+^)-ATPase activity/expression, Herman et al. [49] observed stimulatory effects of PGE_1_ and PGE_2_ on (Na^+^ + K^+^)-ATPase expression/activity in primary cultures of rabbit renal proximal tubule cells. Furthermore, Pöscke et al. [50] showed that PGE_2_ stimulates the renin-angiotensin-aldosterone system, which stimulates (Na^+^ + K^+^)-ATPase activity, leading to sodium and water retention. Because GV sPLA_2_ is involved in the generation of PGE_2_ [11], it is possible that reduced expression/activity of (Na^+^ + K^+^)-ATPase found in *Pla2g5*^*−/−*^ mice could also be due to reduced PGE_2_ levels. Moreover, a previous study from our group showed that high concentrations of albumin decreased the expression and activity of (Na^+^ + K^+^)-ATPase in proximal tubule cells [51]. This observation supports the hypothesis that specific tubular alterations in *Pla2g5*^*−/−*^ mice, including the reduced expression/activity of (Na^+^ + K^+^)-ATPase, are probably due to the increased tubular protein concentration observed in *Pla2g5*^*−/−*^ mice.

Another protective effect of GVsPLA2 in the kidney could result from its action, through PGE_2_ production, in promoting resident immune cells with a suppressive phenotype, such as immune inhibitory dendritic cells (DCs) and regulatory Foxp3^+^ T cells (Tregs). In this regard, Tregs exert protective effects in the kidney, as well as in other organs, against exacerbated and harmful pro-inflammatory responses and acute injury [52–56]. Evidence shows that PGE_2_ is capable of inducing differentiation of naive T cells into regulatory T cells, and suppressive DCs express high levels of COX-2 along with production of IL-10 and TGF-β, cytokines that are important for differentiation into regulatory Foxp3+ T cells (Tregs) [57–59]. The molecular mechanism involves PLA2 binding, with high affinity, to a mannose receptor (CD206) expressed in DCs and macrophages [60]. Mannose receptor activation upregulates COX-2 expression and increases PGE_2_ secretion by these cells [58, 59]. In turn, PGE_2_, via the EP2 receptor in T cells, increases Foxp3 mRNA and protein levels as well as its promoter activity, inducing differentiation of naive T cells into suppressive Foxp3^+^ T cells (Tregs) [57, 58, 61]. This purported protective effect of PLA2 was confirmed in different models of disease in mice, for instance Parkinson disease and cisplatin-induced nephrotoxicity [58, 59]. In a cisplatin-induced acute kidney injury model, treatment with PLA2 attenuated tissue damage by reducing serum creatinine, blood urea nitrogen, production of pro-inflammatory cytokines, such as IL-6 and TNF-α, and macrophage infiltration [59]. The effects of PLA2 were mediated by the binding and activation of the mannose receptor (CD206) in DCs, followed by an increase in PGE_2_ secretion. PGE_2_ induced Treg differentiation and IL-10 production by Tregs and DCs [59]. These IL-10-producing Tregs and DCs exert protective effects in the kidney by reducing monocyte/macrophage infiltration and production of pro-inflammatory cytokines [52–56, 59]. Thus, it is possible that the lack of GVsPLA2 expression, with consequent reduction in local PGE_2_ production, could decrease the suppressive phenotype of resident immune cells in the kidney, facilitating a prone inflammatory environment and changes in renal tissue homeostasis, such as tubular impairment and fibrosis.

Therefore, despite reports showing renal expression of GV sPLA_2_ and the physiologic effects of eicosanoids, its enzymatic products, the function of this particular enzyme on the kidney is not well known. Our results highlight a key role of GV sPLA_2_ in renal homeostasis in the maintenance of tubular cell function and integrity, participating in sodium handling through regulation of cortical (Na^+^ + K^+^)-ATPase expression and activity. Future experiments will further elucidate the division of labor between GV sPLA_2_ and other PLA_2_ enzymes as well as the molecular mechanisms involved in the renal effects.

## Abbreviations

CCr: creatinine clearance
DC: dendritic cell
GIVA: PLA_2_, group IV A phospholipase A_2_
γGT: γ-glutamyl transpeptidase
GV sPLA_2_: group V secreted phospholipase A_2_
FE_Na_^+^: sodium excretion fraction
LDH: lactate dehydrogenase
LT: leukotriene
PAS: periodic acid-Schiff reagent
PG: prostaglandin
PLA_2_: phospholipase A_2_
sPLA_2_: secreted phospholipase A_2_
Treg: regulatory T-cell
WT: wild-type
